# Dietary α-ketoglutarate alleviates glycinin and β-conglycinin induced damage in the intestine of mirror carp (*Cyprinus carpio*)

**DOI:** 10.3389/fimmu.2023.1140012

**Published:** 2023-04-28

**Authors:** Qiaohua Luo, Rendong Qian, Zongsheng Qiu, Fernando Y. Yamamoto, Yingying Du, Xiaowen Lin, Jianhua Zhao, Qiyou Xu

**Affiliations:** ^1^ College of Life Science, Huzhou University, Huzhou, China; ^2^ Nation Local Joint Engineering Laboratory of Aquatic Animal Genetic Breeding and Nutrition, Zhejiang Provincial Key Laboratory of Aquatic Bioresource Conservation and Development Technology, Huzhou, China; ^3^ Thad Cochran National Warmwater Aquaculture Center Agriculture and Forestry Experiment Station, Mississippi State University, Starkville, MS, United States; ^4^ Department of Wildlife, Fisheries and Aquaculture, Mississippi State University, Starkville, MS, United States

**Keywords:** α-ketoglutarate, glycinin, β-conglycinin, intestine immunity, *Cyprinus carpio*

## Abstract

This study investigated the glycinin and β-conglycinin induced intestinal damage and α-ketoglutarate alleviating the damage of glycinin and β-conglycinin in intestine. Carp were randomly divided into six dietary groups: containing fish meal (FM) as the protein source, soybean meal (SM), glycinin (FMG), β-conglycinin (FMc), glycinin+1.0% α-ketoglutarate (AKG) (FMGA), β-conglycinin+1.0% AKG (FMcA). The intestines were collected on 7^th^, and the hepatopancreas and intestines were collected on 56^th^. Fish treated with SM and FMc displayed reduced weight gain, specific growth rate, and protein efficiency. On 56^th^ day, Fish fed on SM, FMG and FMc presented lower superoxide dismutase (SOD) activities. FMGA and FMcA had higher SOD activity than those fed on the FMG and FMc, respectively. In intestine, fish fed on the SM diets collected on 7^th^ presented upregulated the expression of transforming growth factor beta (TGFβ1), AMP-activated protein kinase beta (AMPKβ), AMPKγ, and acetyl-CoA carboxylase (ACC). Fish fed FMG presented upregulated expression of tumor necrosis factor alpha (TNF-α), caspase9, and AMPKγ, while downregulated the expression of claudin7 and AMPKα. FMc group presented upregulated expression of TGFβ1, caspase3, caspase8, and ACC. Fish fed FMGA showed upregulated expression of TGFβ1, claudin3c, claudin7, while downregulating the expression of TNF-α and AMPKγ when compared to fish fed FMG diet. FMcA upregulated the expression of TGFβ1, claudin3c than fed on the FMc. In intestine, the villus height and mucosal thickness of the proximal intestine (PI) and the distal intestine (DI) were decreased and crypt depth of the PI and mid intestine (MI) were increased in SM, FMG and FMc. In addition, fish fed on SM, FMG and FMc presented lower citrate synthase (CS), isocitrate dehydrogenase (ICD), α-ketoglutarate dehydrogenase complex (α-KGDHC) Na^+^/K^+^-ATPase activity in DI. FMGA had higher CS, ICD, α-KGDHC, and Na^+^/K^+^-ATPase activity in PI and MI than those fed on the FMG. FMcA had higher Na^+^/K^+^-ATPase activity in MI. In conclusion, dietary soybean meal destroys the intestine’s health, the adverse effects are related to the presence of β-conglycinin and glycinin, especially glycinin. AKG may regulate intestinal energy *via* tricarboxylic acid cycle, thereby alleviating the damage intestinal morphology caused by the dietary soybean antigen proteins.

## 1 Introduction

Given the increased demand and limited capture of forage fish, fishmeal prices have reached all-time high, with limited supply (1). As an alternative to fishmeal, soybean meal is a plant-based ingredient with high levels of protein, balanced amino acid profile, and other nutrients required by aquatic animals (2). However, soybeanmeal cannot completely replace fishmeal because of the antinutritional factors such as allergens, trypsin inhibitor and saponins (3). High inclusion of soybean meal in fish aquafeeds decreased growth performance in Japanese seabass (*Lateolabrax japonicus*) (4), orange-spotted grouper, and (*Epinephelus coioides*) (5). These adverse effects are often related to the presence of anti-nutritional factors, especially β-conglycinin and glycinin (6). These two antigenic proteins are considered the major cause of reduced growth and increased intestinal inflammation in aquatic animals (7). Previous studies have showed that 4% to 8% β-conglycinin and/or glycinin can damage the structural integrity of the intestine, reduce the immune function and compromise the growth performance of juvenile golden crucian carp (*Carassius auratus*) (8, 9) and grass carp (*Ctenopharyngodon idella*) (10, 11).

In this context, supplementation of functional additives is an important strategy to maintain the intestine health. α-ketoglutaric acid (AKG) is an intermediate for the tricarboxylic acid (TCA) cycle is involved in pleiotropic metabolic and regulatory pathways in the cell, including energy production (12). AKG has the function of improve intestinal immunity and inhibit apoptosis in response to inflammatory stimuli (13), which can ultimately compensate the energy consumption in the intestinal health. In addition, AKG has alleviative effect on oxidative stress as a source of energy and an antioxidant in mammalian cells (14). Similar results were also observed on juvenile red drum (*Sciaenops ocellatus*) (15). Thus, it is hypothesized that AKG could protect fish against glycinin and β-conglycinin injury.

Nevertheless, the alleviating effects of α-ketoglutarate on the damage of glycinin and β-conglycinin in intestinal mucosa of Mirror carp have not been elucidated. Our research group showed that the intestinal inflammation and tight junction protein showed a trend of first serious and then recovery when addition β-conglycinin and Glycinin was 4% to 16%, respectively (16). Therefore, the objective of this study was to investigate the effects of high-lever glycinin and β-conglycinin on growth performance, antioxidant capacity, intestinal health and energy of mirror carp (*Cyprinus carpio*), and evaluated whether AKG supplementation had potential alleviating effects on the glycine and β-conglycine induced injuries in the intestinal mucosa.

## 2 Materials and methods

### 2.1 Experimental diets

Glycinin (purity, 85%) and β-conglycinin (purity, 88%) were provided by the China Agricultural University. α-ketoglutarate (purity, 98%) was purchased from Sigma-Aldrich. For this study, six experimental diets were formulated to be isonitrogenous (33%) and isolipidic (6%). The ingredients and nutrient composition of all diets are shown in **Table 1**. For the control group (FM), fish meal was used as the main protein source, and the other five diets were formulated with either 56.0% soybean meal (SM), 22.8% glycinin (FMG), 22.0% β-conglycinin (FMc), 22.8% glycinin + 1.0% α-ketoglutarate (FMGA), or 22.0% β-conglycinin + 1.0% α-ketoglutarate (FMcA). Dietary ingredients were ground into fines to pass through a 60 mesh sieve. Homogenized ingredients were thoroughly mixed with fish oil, soybean oil, phospholipid, and water, and mixtures were extruded (twin screw extruded F-26, South China University of Technology, Guangzhou, China), using a 1.5 mm die-plate. The diets were dried in a ventilated oven at 40 °C, until reached a constant dry matter, and stored at -20 °C until feeding.

**Table 1:** Formulation of the experimental diets fed to Mirror carp (*Cyprinus carpio*), and their analyzed nutrient composition.

Ingredients	Groups					
	FM	SM	FMG	FMc	FMGA	FMcA
Fish meal	38.00	5.00	5.00	5.00	5.00	5.00
Soybean meal	0.00	56.00	0.00	0.00	0.00	0.00
Glycinin	0.00	0.00	22.80	0.00	22.80	0.00
β-conglycinin	0.00	0.00	0.00	22.00	0.00	22.00
α-ketoglutarate	0.00	0.00	0.00	0.00	1.00	1.00
Wheat middling	49.90	28.72	57.10	57.80	56.10	56.80
Cellulose	3.60	0.00	3.65	3.69	3.65	3.69
Fish oil	0.00	3.20	3.20	3.20	3.20	3.20
Soybean oil	3.10	0.30	1.40	1.40	1.40	1.40
Soybean lecithin oil	1.00	1.00	1.00	1.00	1.00	1.00
Vitamin premix^a^	0.50	0.50	0.50	0.50	0.50	0.50
Mineral premix^b^	0.20	0.20	0.20	0.20	0.20	0.20
Choline chloride	0.30	0.30	0.30	0.30	0.30	0.30
Ca(H_2_PO_4_)_2_	1.20	2.00	2.00	2.00	2.00	2.00
Carboxymethyl cellulose	2.00	2.00	2.00	2.00	2.00	2.00
Lysine	0.10	0.23	0.00	0.00	0.00	0.00
Methionine	0.10	0.51	0.65	0.65	0.65	0.65
L-Threonine	0.00	0.04	0.20	0.26	0.20	0.26
Proximate analysis (%)						
Moisture	5.70	5.90	5.87	6.30	5.88	5.25
Crude protein	33.36	34.08	33.62	33.42	33.59	33.75
Crude lipid	6.50	6.35	6.48	6.42	6.40	6.34
Ash	11.52	7.41	4.54	4.73	4.66	4.61

a The premix provided the following per kg of diets: A 8 000IU, VC 500mg, VD 3 000IU, VE 60mg, VK 35mg, VB_1_ 15mg, VB_2_ 30mg, VB_6_ 15mg, VB_12_ 0.5mg;

b The premix provided the following per kg of diets:FeSO_4_•H_2_O 960 mg, CuSO_4_•5H_2_O 12 mg, MnSO_4_•H_2_O 33 mg, ZnSO_4_•H_2_O 70 mg, Na_2_SeO_3_ 1.2 mg, Ca(IO_3_)_2_ 1.4mg, CoCl_2_•6H_2_O 2.4 mg, Zeolite meal 920 mg.

### 2.2 Feeding management

The feeding trial was carried out at the College of Life Science, Huzhou University. Prior commencement of the feeding trial, fish were acclimated in the recirculating system for two weeks. A total of 450 carps of similar sizes (mean initial body weight 2.08 ± 0.06 g) were randomly distributed into six treatments, 3 replicate tanks per treatment, with 25 fish per tank (500 L water). Fish were fed to apparent satiation thrice daily (08:00, 13:00, and 18:00) for 8 weeks; and uneaten feed was collected after 45 min, dried and weighed to record feed intake (17). During the experimental period, the carp were cultured in a recirculating aquaculture system with continuous aeration, with 1/3 of the water being changed every day. The water temperature ranged from 24.6 to 30.4°C, dissolved oxygen was no less than 6.0 mg l^-1^, and the ammonia nitrogen concentrations was less than 0.3 mg L^-1^.

### 2.3 Growth performance

At the beginning and end of the trial, fish in each tank were group weighed and counted to calculate growth performance, including Weight gain (WG %), Specific growth rate (SGR), Survival rate (SR %), PE (Protein efficiency %), Feed conversion ratio (FCR) (18). The production performance were calculated as follows:

Weight gain (WG, %) = (FBW−IBW)/IBW×100;


Specific growth rate (SGR, %/d) = [ln(FBW)−ln(IBW)]×100 /d;

$$Survival\;rate\;(SR,\;\%)\;=\;N_{t}/N_{0}\times 100;$$

Protein efficiency (PE, %) = (FBW−IBW)/(F×P)×100;

Feed conversion ratio (FCR) =F/(FBW − IBW);

Abbreviations: Number of initial fish, N_0_; number of final fish, N_t_; initial body weight, IBW; final body weight, FBW; Feeding days, d; total diet intake, F; feed crude protein content, P.

### 2.4 Sample collection and analysis

Intestine samples collected on the 7^th^ day were dissected quickly on ice. 3 fish was pooled as one sample, and six samples per treatment. The intestine samples were stored at -80°C to analyze the relative gene expression during a short-term exposure to the experimental feed.

On the 56^th^ day, after weight and length were measured, three fish from each tank (nine per treatment). The fish were then dissected to obtain the hepatopancreas and intestine quickly on ice. The hepatopancreas stored at -80°C for antioxidant capacity analysis. The intestinal tract was immediately divided into proximal intestine (PI), mid intestine (MI), and distal intestine (DI), stored at -80°C for key enzymes in tricarboxylic acid cycle and Na^+^/K^+^-ATPase analysis. An additional segment of PI, MI, and DI was fixed using Bouin solution for histology.

### 2.5 Antioxidant status

Glutathione (GSH, Cat. No. A006-2-1), malondialdehyde (MDA, Cat. No. A003-1) content; the activities of total superoxide dismutase (SOD, Cat. No. A001-1), and catalase (CAT, Cat. No.A007-1-1) were determined using the commercial kits purchased from Nanjing Jiancheng Bioengineering Institute.

### 2.6 Gene expression analysis

The total RNA was extracted from the proximal intestine, mid intestine, and distal intestine by Rapid Extraction Kit extracted total RNA (RN28; Aidlab Bio Inc., Beijing, China). RNA concentration and purity were measured by the spectrophotometry analysis (A260:A280 nm ratio) within the ratio specified by the kit (1.8-2.2). Subsequently, cDNA was synthesized by a MonScript™ RTIII All-in-One Mix (MR05001M; Monad Bio Inc., Wuhang, China). The MonAmp™ SYBR^®^ Green qPCR Mix (MQ10101S; Monad Bio Inc., Wuhang, China) used mRNA level analysis by quantitative real-time PCR on a CFX-96 Real-Time PCR Detection System (Bio-Rad Laboratories, Inc., USA). The reactions were done in a volume of 20 μL containing 0.4 μL of 10 μM each of reverse and forward primers, 1 μL of diluted cDNA, 10 μL of MonAmp™ SYBR^®^ Green qPCR Mix, and 8.2 μL of Nuclease-Free Water. PCR conditions were as follows: 95 °C for 5 min followed by 40 cycles of 95 °C for 10 s, 60°C for 10 s and 72 °C for 30 s. The online Primer3 (http://primer3.ut.ee) software was used to design the primers. Target gene mRNA relative expression levels were calculated using the 2^-ΔΔCt^ method and corrected for the expression of the normalizing gene β-actin, according to Luo et al. (19). The primers used for quantitative fluorescence analysis are shown in **Table 2**.

**Table 2:** Primer sequences of target genes from mirror carp (*Cyprinus carpio*), to measure the relative expression of the genes through quantitative real-time PCR.

Target genes	Primer sequence (5’-3’)	Gene ID
TNF-α^a^	AAGTCTCAGAACAATCAGGAA TGCCTTGGAAGTGACATT	XM_019088899.2
TGFβ1^b^	ACACGGTCACTTTGGTGTCA CAATGTGGGTTGCAGAACAG	XM_019072371.2
IL-1β^c^	AACTTCACACTTGAGGAT GACAGAACAATAACAACAAC	XM_019080073.2
claudin3c	GCACCAACTGTATCGAGGATG GGTTGTAGAAGTCCCGAATGG	XM_042710912.1
claudin7	CTTCTATAACCCCTTCACACCAG ACATGCCTCCACCCATTATG	XM_042732468.1
occludin	ATCGGTTCAGTACAATCAGG GACAATGAAGCCCATAACAA	KF975606.1
caspase3	CTCTACGGCACCAGGTTACTACTC GCCATCATTTCACAAAGGGACT	XM_019110173.2
caspase8	AAGCTCCTCATTGAAGAACCG ATCGTCCTGAACCACAACCTC	XM_042759183.1
caspase9	GCAAGCCCAAACTGTTCTTCAT CGTCCATCTGGTCATCTATCCC	XM_042750058.1
AMPKα^d^	GATGCCCTCTGGATGCTCTC GATGTCGTATGGTTTGCTCTGG	XM_042777568.1
AMPKβ^e^	AAACCTGAGGAACGCTTCAA CATCACATGGTTGGGTTCTG	XM_042711618
AMPKγ^f^	TGGGACAACAAACTGCAGAG GCAAGGGAATGAAGGAAACA	XM_042716989.1
ACC^g^	GTCACTGGCGTATGAGGATATT TCCACCTGTATGGTTCTTTGG	XM_042724983.1
TOR^h^	ATCTACGGCAAGACGAGAGG GTTGGTGGAGAGTGGGATCA	XM_042761449.1
4E-BP^i^	GGTGCTGATCAACGAGTCAA CAGGACGTTCTTGCTTGTCA	XM_042737175.1

a TNF-α, tumor necrosis factor alfa;

b TGF-β1, transforming growth factor beta;

c IL-1β, interleukin-1 beta;

d AMPKα, AMP-activated protein kinase alfa;

e AMPKβ, AMP-activated protein kinase beta;

f AMPKγ, AMP-activated protein kinase gamma;

g ACC, acetyl-CoA carboxylase;

h TOR, target of rapamycin;

i 4E-BP, eIF4E-binding protein.

### 2.7 Histology of the intestine

The 1 cm segments were cut from the PI, MI, and DI. The 1 cm intestinal segments were processed, embedded, and stained according by Hangzhou Haoke Biotechnology Co., Ltd. Randomly selected fields were observed and pictures were taken by a camera coupled with a light microscope (40×). The images were measured and calculated for villus height, crypt depth, mucosal thickness according to the method previously described by Lin et al. (20) using the software Adobe Photoshop 2021. The average value was calculated from the measurement results from each sample index.

### 2.8 Key enzymes in tricarboxylic acid cycle and Na^+^/K^+^-ATPase in the intestine

Citrate synthase (CS, Cat. No. MM-204201), Isocitrate dehydrogenase (ICD, Cat. No. MM-3280701), α-ketoglutarate dehydrogenase complex (α-KGDHC, Cat. No. MM-9176301), and Na^+^/K^+^-ATPase (Cat. No. MM-166201), were determined by the enzyme-linked immunosorbent assays (ELISA), following the manufacturer’s procedures (Jiangsu Meimian Industrial Co., Ltd., China)

### 2.9 Statistical data analysis

All statistical analyses were performed using SPSS, version 26.0 software. The level of significance was set to *P*< 0.05. Data were subjected to the one-way analysis of variance (ANOVA) followed by Tukey’s test was performed for comparison of means, and data is presented as means ± standard error (SE).

## 3 Results

### 3.1 Growth performance

**Figure 1:**
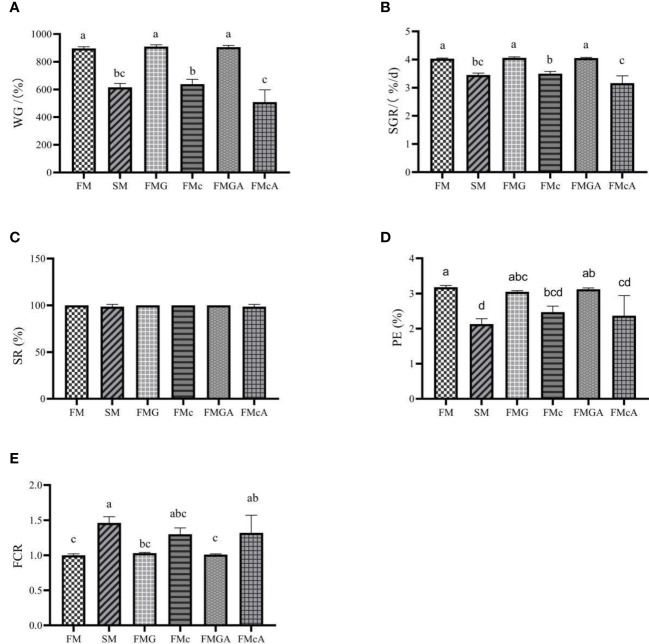
Growth performance of mirror carp at different treatments fed with dies containing fishmeal (FM), soybean meal (SM), glycinin (FMG), B-conglycinin (FMc), AKG+glycinin (FMGA), AKG+B-conglycinin (FMcA) after eight weeks. **(A)** WG, weight gain (%); **(B)** SGR, specific growth rate (%/day); **(C)** SR, survival rate (%); **(D)** PE, protein effiency (%); **(E)** FCR, feed conversion ratio; Data are presented as mean + SE, (n = 3). ^a, b, c^ Mean values with different letters were significantly different (P<0.05).

The effect of soybean antigen proteins and α-ketoglutarate on growth performance of mirror carp is shown in **Figure 1**. Compared with the FM group, there were no significant differences in SR. The WG, SGR, and PE were significantly reduced in SM and FMc group. In addition, the FCR was significantly increased in SM. Feeding the fish with the FMG diets did not affect significantly WG, SGR, SR, PE, and FCR.

Feeding the fish with the FMGA diets did not affect significantly WG, SGR, SR, PE, and FCR, when compared to the fish fed FMG diets. Feeding the fish with the FMcA diets did not affect significantly SR, PE, and FCR, but WG and SGR were significantly decreased, when compared to the fish fed FMc diets.

### 3.2 Antioxidant capacity in the hepatopancreas

**Figure 2:**
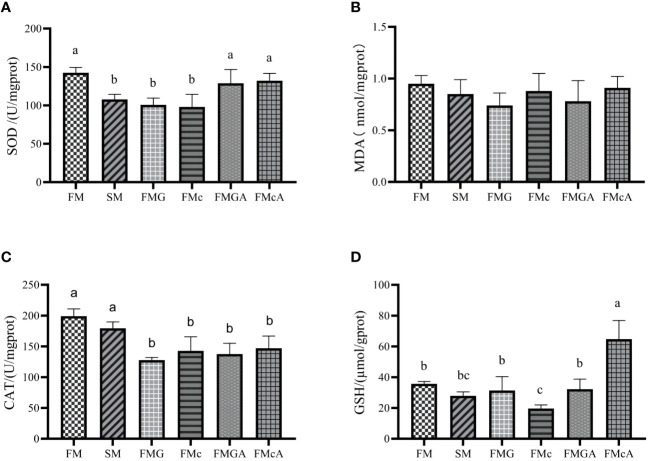
Antioxidative ability in the hepatopancreas of mirror carp at different treatment fed with diets containing fishmeal (FM), soybean meal (SM), glycinin (FMG), B-conglycinin (FMc), AKG+B-conglycinin (FMcA) after eight weeks. **(A)** T-SOD, total superoxide dismute (U/mg prot); **(B)** MDA, malondialdehyde (mmol/mg prot). **(C)** CAT, catalase (U/mg prot); **(D)** GSH, glutathione (umol/gprot); Data are presented as mean +SE, (n = 6) ^a, b, c^ Mean values with different letters were significantly different (P<0.05).

The effect of soybean antigen proteins and α-ketoglutarate on antioxidative ability in the hepatopancreas was given in **Figure 2**. Compared with the FM group, there was no significant change in MDA content, however, SOD activity were significantly decreased in SM, FMG and FMc group; the CAT activity in FMG and FMc group and the GSH content in FMc group were decreased significantly.

Compared with the FMG group, there were no significant change in GSH content, MDA content, and CAT activity. However, the SOD activity of FMGA group increased significantly. Compared with the FMc group, there was no significant change in MDA content, CAT activity. However, the GSH content, SOD activity of FMcA group increased significantly.

### 3.3 Relative expression levels of inflammatory cytokines genes in the intestine

**Figure 3:**
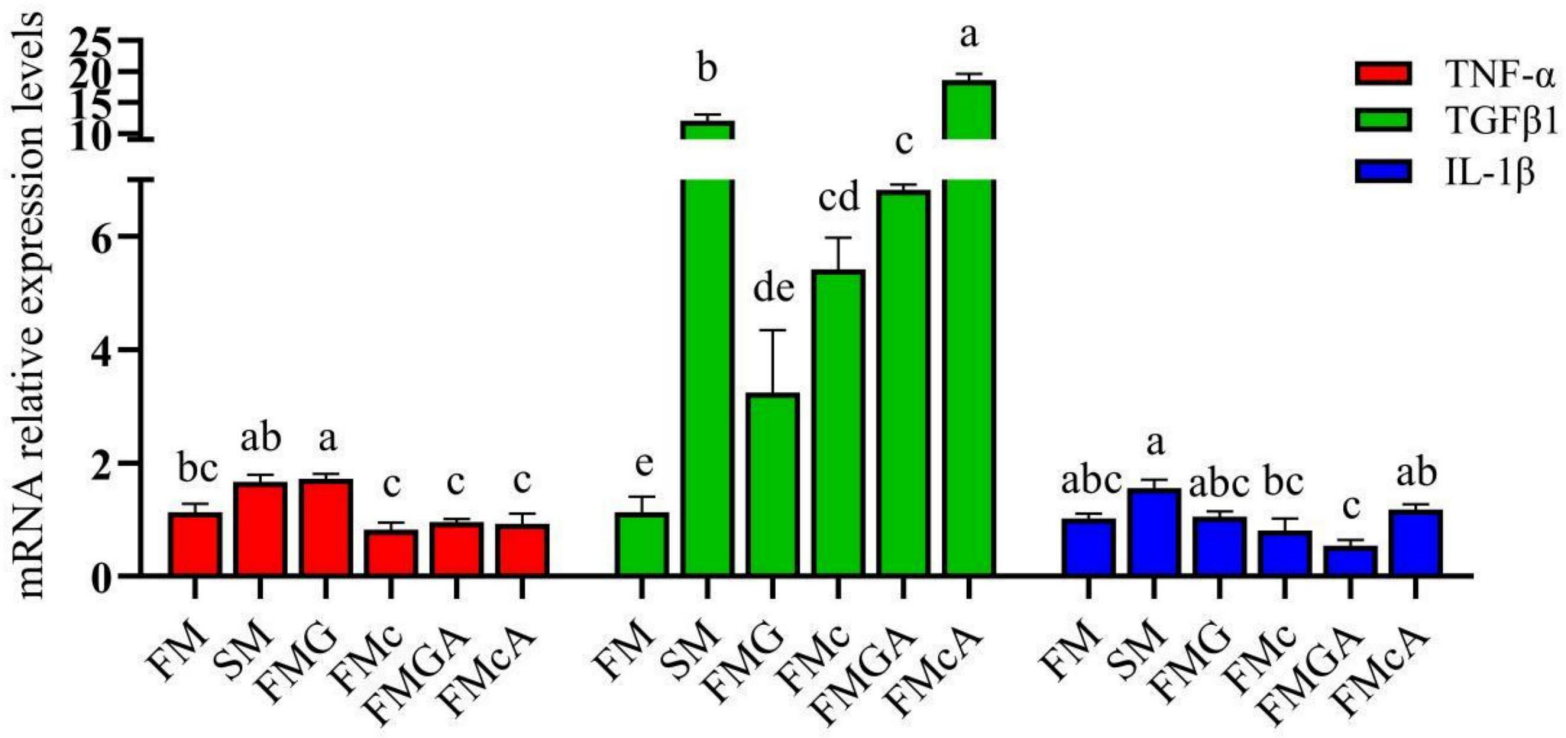
Relative expression levels of inflammatory cytokins in the intestine of mirror carp at different treatments fed with diets containing fishmeal (FM), soybeans meal (SM), glycinin (FMG), B-conglycinin (FMc), AKG+B-conglycinin (FMcA) after eight weeks. TNF-a, tumor necrosis factor alfa; TGF-B1, transforming growth factor beta; IL-1B, interleukin-1 beta. Data are presented as means + SE, (n = 3). ^a, b, c^ Mean values with different letters were significantly different (P<0.05).

The effect of soybean antigen proteins and α-ketoglutarate on the mRNA levels of inflammatory cytokines genes in the intestines are shown in **Figure 3**. Compared with the FM group, the mRNA expression levels of TNF-α and IL-1β were not significantly different, but the mRNA expression levels of TGFβ1 was significantly increased in SM and FMc group; the mRNA expression levels of TGFβ1, IL-1β were not significantly different, but the mRNA expression levels of TNF-α was significantly increased in FMG group.

Compared with the FMG and FMc group respectively, the mRNA level of IL-1β was not significantly different in FMGA and FMcA group, but the mRNA expression levels of TGFβ1 were significantly increased in FMG and FMcA group, respectively.

### 3.4 Relative expression levels of junction protein in the intestine

**Figure 4:**
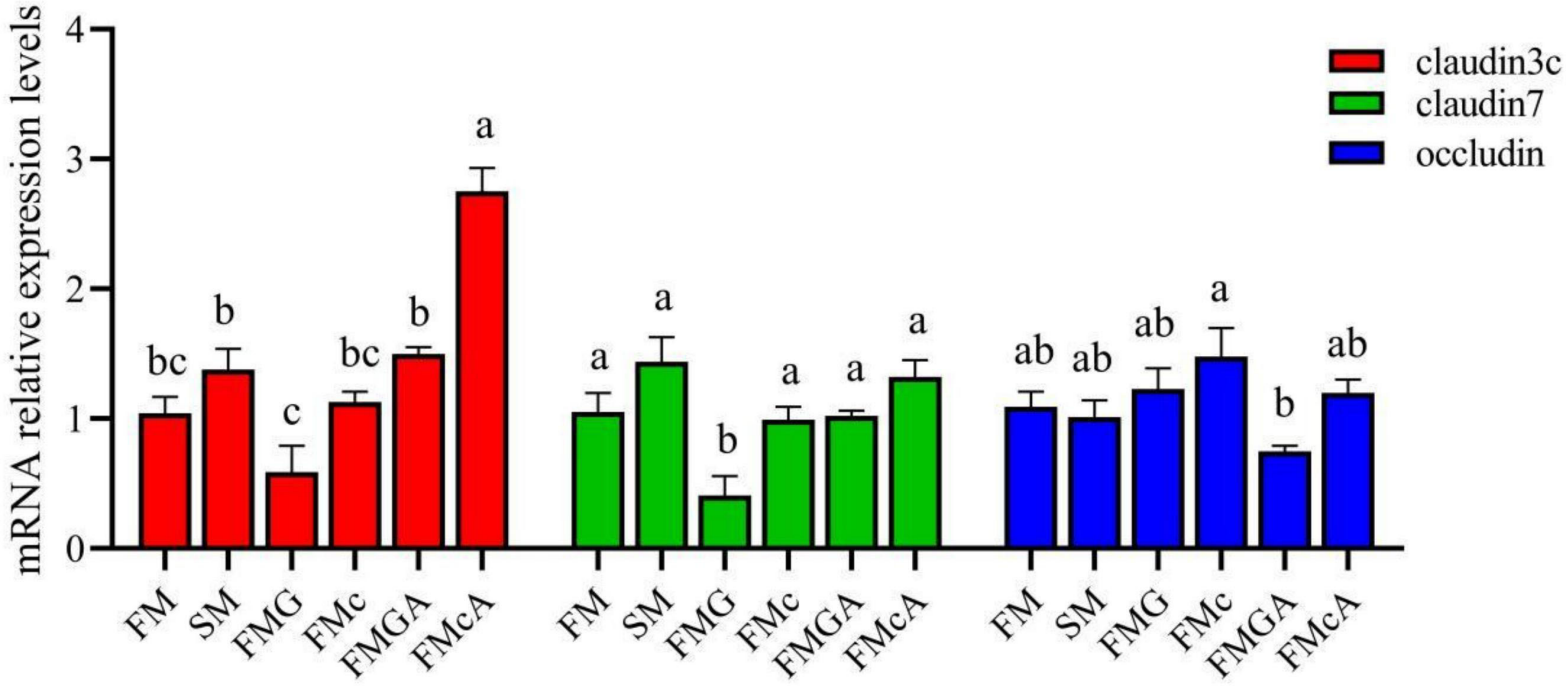
Relative expression levels of junction protein in the intestine of mirror carp at different treatment fed with the diets containing fishmeal (FM), soybean meal (SM), glycinin (FMG), B-conglycinin (FMcA)< AKG+B-conglycinin (FMGA), AKG+B-conglycinin (FMcA) after eight weeks. Data are presented as means + SE, (n = 3). ^a, b, c^ Mean values with different letters were significantly different (P<0.05).

The effect of soybean antigen proteins and α-ketoglutarate on the mRNA levels of junction protein in the intestines are shown in **Figure 4**. Compared with the FM group, the mRNA expression levels of claudin3c, claudin7, and occludin were not significantly different in SM and FMc group; the mRNA expression levels of claudin3c and occludin were not significantly different, but the mRNA expression levels of claudin7 was significantly decreased in FMG group.

Compared with the FMG group, the mRNA level of occludin was not significantly different, but the mRNA expression levels of claudin3c and claudin7 were significantly increased in FMGA group. Compared with the FMc group, the mRNA level of occludin and claudin7 were not significantly different, but the mRNA expression levels of claudin3c was significantly increased in FMcA group.

### 3.5 Relative expression levels of apoptosis factors in the intestine

**Figure 5:**
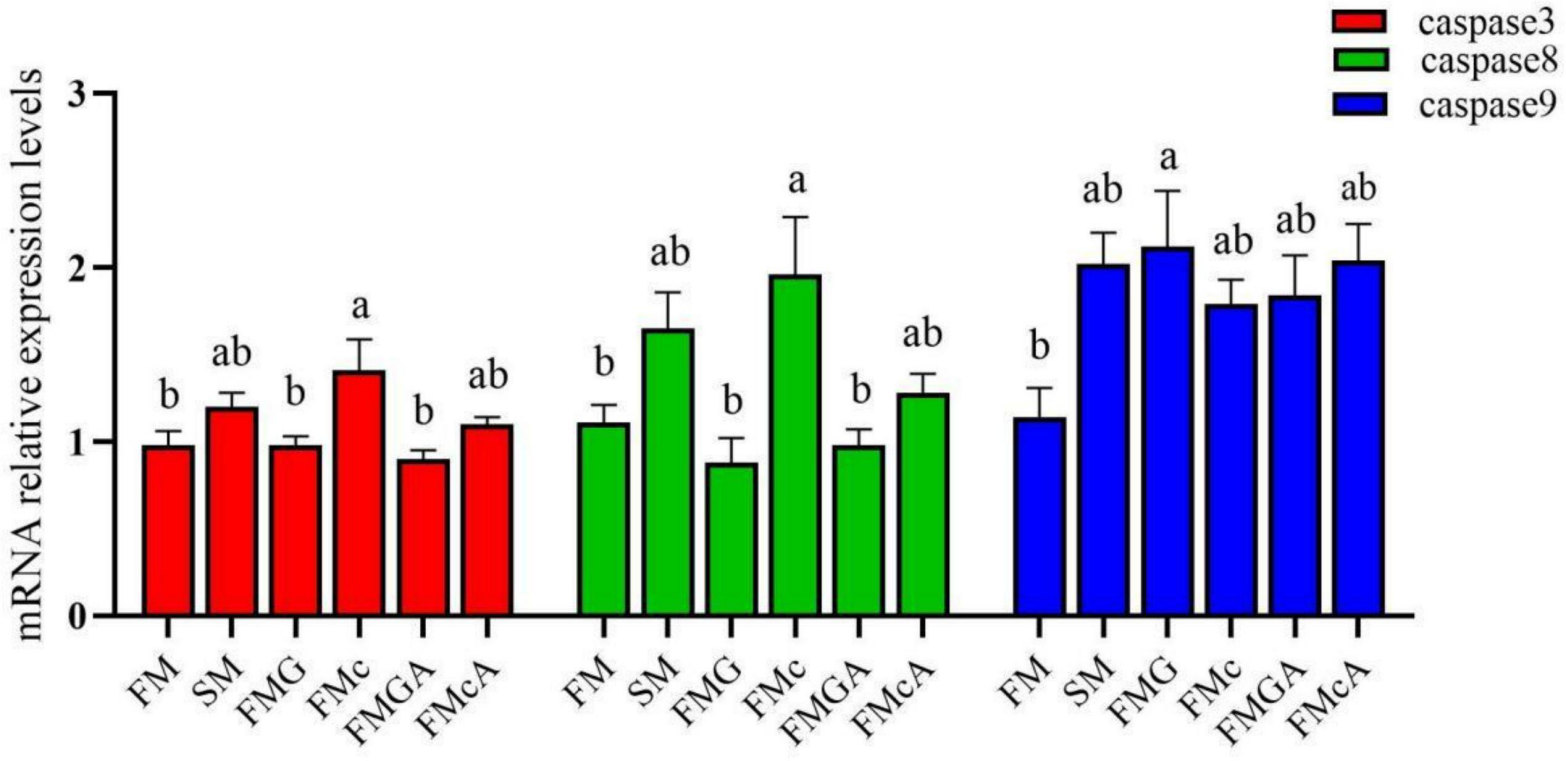
Relative expression levels of apoptosis factors in the intestine of mirror carp at different treatments fed with diets containing fishmeal (FM), soybean meal (SM), glycinin (FMG), B-conglycinin (FMc), AKG+ glycinin (FMGA), AKG+B-conglycinin (FMcA) after eight weeks. Data are presented as means + SE, (n = 3). ^a, b, c^ Mean values with different letters were significantly different (P<0.05).

The effect of soybean antigen proteins and α-ketoglutarate on the mRNA levels of apoptosis factors in the intestines was given in **Figure 5**. Compared with the FM group, the mRNA expression levels of caspase3, caspase8, and caspase9 were not significantly different in SM group. In FMG group, the mRNA expression levels of caspase3 and caspase8 were not significantly different, but the mRNA expression levels of caspase9 was significantly increased; the mRNA expression levels of caspase9 was not significantly different, but the mRNA expression levels of caspase3 and caspase8 were significantly increased in FMc group.

Compared with the FMG and FMc group, the mRNA expression levels of caspase3, caspase8, and caspase9 were not significantly different in FMGA and FMcA group.

### 3.6 Relative expression levels of AMPK/ACC and TOR signaling pathway in the intestine

**Figure 6:**
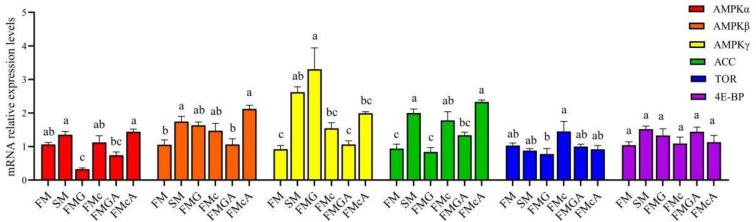
Relative expression levels of AMPK/ACC and TOR signaling pathways in the intestine of mirror carp at different treatments fed with diets containing fishmeal (FM), soybean meal (SM), glycinin (FMG), B-conglycinin (FMc), AKG+ glycinin (FMGA), AKG+B-conglycinin (FMcA) after eight weeks. AMPKa, AMP-activated protein kinase gamma; ACC, acetyl-CoA carboxylase; TOR, target of rapamycin; 4E-BP, eIF4E-binding protein. Data are presented as means + SE, (n = 3). ^a, b, c^ Mean values with different letters were significantly different (P<0.05).

The effect of soybean antigen proteins and α-ketoglutarate on the mRNA levels of AMPK/ACC and TOR signaling pathway in the intestines was given in **Figure 6**. Compared with the FM group, the mRNA expression levels of AMPKα, target of rapamycin (TOR), and eIF4E-binding protein (4E-BP) were not significantly different, but the mRNA level of AMPKβ, AMPKγ, and acetyl-CoA carboxylase (ACC) were significantly increased in SM group; the mRNA expression levels of AMPKβ, ACC, TOR, and 4E-BP were not significantly different, but the mRNA level of AMPKγ were significantly increased and the mRNA level of AMPKα were significantly decreased in FMG group; the mRNA expression levels of AMPKα, AMPKβ, AMPKγ, TOR and 4E-BP were not significantly different, but the mRNA level of ACC was significantly increased in FMc group.

Compared with the FMG group, the mRNA expression levels of AMPKα, AMPKβ, ACC, TOR, and 4E-BP were not significantly different, but the mRNA level of AMPKγ was significantly decreased in FMGA group. Compared with the FMc group, the mRNA expression levels of AMPKα, AMPKβ, AMPKγ, ACC, TOR and 4E-BP were not significantly different.

### 3.7 Morphology of the intestine

**Figure 7:**
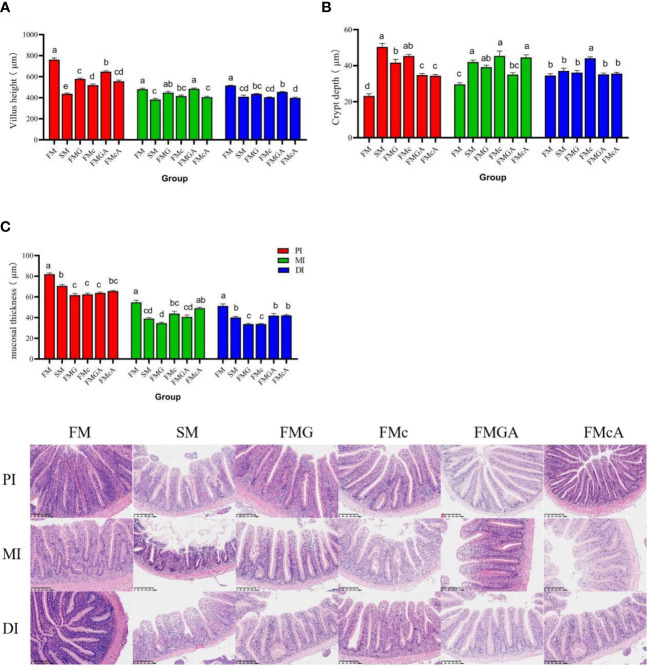
Morphology of the intestines of mirror carp at different treatments fed with diets containing fishmeal (FM), soybean meal (SM)m glycinin (FMG), B-conglycinin (FMc), AKG+ glycinin (FMGA), AKG+B-conglycinin (FMcA) after eight weeks. PI, proximal intestine; MI, mid intestine; DI, distal instestine. **(A)** villus height (um); **(B)** crypt depth (um); **(C)** mucosal thickness (um). Data are presented as means + SE, (n = 3). ^a, b, c^ Mean values with different letters were significantly different (P<0.05).

The effect of soybean antigen proteins and α-ketoglutarate on morphology of the intestines was given in **Figure 7**. The fish fed on the SM diet, there were no significant change in crypt depth in DI; but the crypt depth were significantly increased in PI and MI. The villus height and mucosal thickness were significantly decreased in PI, MI, and DI. The fish fed on the SM diet, there were no significant change in villus height (MI) and crypt depth (DI); but the villus height (PI and DI) and mucosal thickness (PI, MI, and DI) were significantly decreased and crypt depth (PI and MI) were significantly increased. The fish fed on the FMc diet, the villus height and mucosal thickness were significantly decreased and crypt depth were significantly increased in PI, MI, and DI.

Compared with the FMG group, there were no significant change in villus height, crypt depth (MI and DI), mucosal thickness (PI and MI), but crypt depth (PI) was significantly decreased and villus height (PI), mucosal thickness (DI) was significantly increased in FMGA group. Compared with the FMc group, there were no significant change in villus height (PI, MI and DI), crypt depth (MI), mucosal thickness (PI and MI), but crypt depth (PI and DI) was significantly decreased and mucosal thickness (DI) was significantly increased.

### 3.8 Key enzymes in tricarboxylic acid cycle and Na^+^/K^+^-ATPase in the intestine

**Figure 8:**
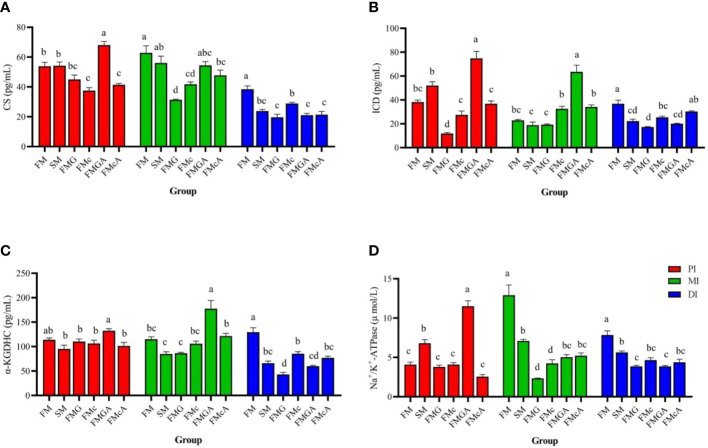
Key enzymes in the tricarboxylic acid cycle and Na^+^/K^+^-ATPase in the intestine fed with diets containing fishmeal (FM), soybean meal (SM), glycinin (FMG). B-conglycinin (FMc), AKG+ glycinin (FMGA) AKG+B-conglycinin (FMcA) after eight weeks. PI, proximal intestine; MI, mid intestine; DI, distal intestine. **(A)** CS, Citrate synthase; **(B)** ICD, Isocitarte dehydrogenase; (pg/mL) **(C)** a-KGDHC, a-ketaglutarte dehydrogenase complex (pg/mL); **(D)** Na+/K+-ATPase (umol/L). Data are presented as means +SE, (n = 3). ^a, b, c^ Mean values with different letters were significantly different (P<0.05).

The effect of soybean antigen proteins and α-ketoglutarate on key enzymes in tricarboxylic acid cycle and Na^+^/K^+^-ATPase in the intestines was given in **Figure 8**. Compared with the FM group, there were no significant change in CS, ICD, and α-KGDHC (PI and MI) activity, but Na^+^/K^+^-ATPase (PI) was significantly increased and α-KGDHC, ICD, CS (DI), and Na^+^/K^+^-ATPase (MI and DI) activity were significantly decreased in SM group; there were no significant change in CS, Na^+^/K^+^-ATPase (PI), ICD (MI), and α-KGDHC (PI and MI) activity, but CS, Na^+^/K^+^-ATPase (MI and DI), ICD (PI and DI), α-KGDHC (DI) were significantly decreased in FMG group; there were no significant change in ICD, α-KGDHC (PI and MI), and Na^+^/K^+^-ATPase (PI) activity, but CS (PI, MI and DI), α-KGDHC, ICD (DI), Na^+^/K^+^-ATPase (MI and DI) were significantly decreased in FMc group.

Compared with the FMG group, there were no significant change in CS, ICD, α-KGDHC and Na^+^/K^+^-ATPase (DI) activity, but CS, α-KGDHC, ICD and Na^+^/K^+^-ATPase (PI and MI)activity were significantly increased in FMGA group. Compared with the FMc group, there were no significant change in CS (PI and MI), ICD, α-KGDHC (PI, MI, and DI), and Na^+^/K^+^-ATPase (PI and DI) activity, but Na^+^/K^+^-ATPase (MI) activity were significantly increased and CS (DI) activity was significantly decreased in FMcA group.

## 4 Discussion

In the present study, carp fed diets with soybean meal presented lower WG, SGR, and PE than in the control, which is corroborated by the findings of other studies investigating dietary soybean meal (21, 22). Dietary soybean meal increased the FCR, implying that the growth inhibition can be partly attributed to low feed efficiency by adding soybean meal (23). Glycinin and β-conglycinin are the main anti-nutritional factors in soybean meal, which can also adversely affect the growth performance of aquatic animals (3). In this study, dietary β-conglycinin reduced growth performance. This finding is consistent with studies in golden crucian carp (8). β-conglycinin cannot be digested and broken down into small molecules, and it is known to cause allergic reaction in intestinal tract, destruction of intestinal integrity, dysfunction of digestion and absorption of nutrients, inflammation, and oxidative damage, and finally suppressed fish growth performance (24). It was surprising to observe that growth performance was not influenced by the dietary Glycinin. These striking differences in growth performance between these two anti-nutrients may happen due to their functional properties. Glycinin has a higher content of sulfur-containing amino acids than β-conglycinin (25). The composition of essential amino acids is more balanced, so it can better meet the body ‘s intake of essential amino acids. After adding the same dose of soybean antigen proteins, β-conglycinin could reduce the growth performance of carp (26), while glycinin did not affect the growth performance of turbot (27). Moreover, in this study fish were fed to apparent satiation (~9% of their body weight/day), and this high feed intake may have met the requirement of the growth performance of carps, which can be observed on the growth performance. Dietary AKG supplementation can promote growth performance by enhancing the activities of the antioxidant defense system (28). The supplementation of 1.0% AKG diet has also presented positive effects on growth performance of juvenile hybrid sturgeon (*Acipenser schrenckii ♀ × A. baerii ♂*). However, in this study, 1.0% AKG diet has not positive on the WG and SGR of carp, similar results were also observed in juvenile red drum (15). This lack of production performance may have been caused by the physiological differences among these fish species, breeding environments, growth phases, and dietary nutrient levels.

It is widely accepted that inflammation and oxidative stress usually occur simultaneously (29).Various stress factors may cause an imbalance between pro-oxidant and antioxidant systems, which will lead to oxidative stress. MDA, SOD and CAT were used as oxidative stress biomarkers (30). The concentration find in their tissues can reflect their antioxidant capacity (31) The oxidative stress induced by soybean antigen proteins, as evidenced by the depressed levels of SOD and CAT, indicates that glycinin and β-conglycinin can cause SBM-induced oxidative stress in carp. These deleterious effects may be caused by the strong immunogenicity induced by soybean antigen proteins (32). Our results on the elevated activity of SOD suggest that AKG may suppress oxidative damage by glycinin and β-conglycinin. Besides these set of enzymes, GSH is one of the main non-enzymatic antioxidants that protect cells against oxidative damage (33), and it is protects many kinds of cells in body (34). AKG can be converted into glutamine by glutamate dehydrogenase and glutamine synthetase, which can be a marker of antioxidative function (14). It is evident that AKG can improve antioxidative capacity by increasing available glutamine and aiding antioxidative systems (14). Fish fed AKG supplemented feed presented an improvement of antioxidant enzyme levels, including GSH and SOD, indicating its protective efficacy (35). It has also been reported that AKG can positively alleviate the oxidative stress damage in cells, ultimately contributing to cell redox homeostasis (14), and also serve as an energy donor and antioxidant agent *via* the TCA cycle (36).The prooxidant effect of AKG seems to be due to the altered intensity of the TCA cycle in which AKG is preferentially metabolized (37). These data demonstrate that 1.0% AKG administration can alleviate the oxidative stress caused by the dietary soybean antigen proteins *via* the TCA cycle.

After feeding, soybean antigenic protein enters the intestine, destroying the structural stability of the intestine, activating the immune system and causing intestinal inflammation (38). TNF-α plays an essential role in mediating the inflammatory, regulating immune function and other pathophysiological reactions (39). IL-1β is a pleiotropic inflammatory cytokine and plays an important role in the inflammatory process (40). Both TNF-α and IL-1β are important pro-inflammatory factors (41). Intestinal inflammation is related to elevated pro-inflammatory cytokines (42). Enteritis is aggravated by up-regulating pro-inflammatory cytokines (43). In the present study, the expression of pro-inflammatory factor (TNF-α) was upregulated by dietary FMG group, which revealed the occurrence of glycinin-induced enteritis. Similarly, the phenomenon of glycinin-induced enteritis has also been observed in juvenile *Rhynchocypris lagowskii* (44). In addition, the mRNA level of TNF-α was decreased by dietary FMGA group. AKG can improve intestinal immunity and promotes intestinal health (45). Previously, Zhang et al. (34) found that glutamine suppressed TNF-α concentration. It is further confirmed by our study that AKG as a precursor for the biosynthesis of glutamine, increasing intestinal immunity. TGF-β is considered to be important anti-inflammatory cytokines that can inhibit inflammation (46). TGF-β has also played an important role in gut wound repair by promoting epithelial reconstitution through up-regulation of epidermal growth factor (47). Compared with the FMG/FMc group, the mRNA level of TGF-β1 was increased by dietary AKG. These data demonstrate that 1.0% AKG administration can alleviate the intestinal inflammation caused by the dietary soybean antigen proteins.

The intestinal epithelial barrier is the first line of defense against pathogenic microorganisms and toxins in the intestines (48). The connection between intestinal epithelial cells mainly relies on the TJ (49), and mainly composed of occludin, claudins, and others (50), which forms a highly selective barrier and protect animal health. In our study, we found that the mRNA levels of claudin7 was suppressed by glycinin. Claudins are the major barrier-forming proteins of tight junction structure (51). Decreased gene expression of TJ usually represents the weakened intestinal barrier function (52). Similarly, Zhao et al. (53) reported that changes in TJ expression induced by soybean antigen protein lead to intestinal epithelial barrier dysfunction. It demonstrates that glycinin increase intestinal paracellular permeability, decrease tight junction proteins’ expressions, suggesting that glycinin induce intestinal tight junction barrier dysfunction. The result in this study is supported by the findings in carp (54). This might be due to the fact that glycinin, as a foreign antigen, caused immune injury and inflammation, and then inflammatory factors destroyed intestinal epithelial tissue proteins (32). A lot of studies have proved that the impaired intestinal barrier is directly related to the intestinal inflammation (55, 56) and attenuate gut injury through an anti-inflammatory role (57). In the study, the down-regulation of claudin3c, claudin7 observed after dietary soybean antigen protein was abolished by AKG. These observations were partly attributed to AKG supplementation improving glutamine synthesis activity and concentrations of glutamine and glutamic acid in the intestines (58). Dietary alanyl-glutamine in soy protein diets elevated the expression of TJ proteins (59). A number of studies have were reported that dietary glutamine enhance gut development and regulate intestinal barrier function in multiple animal models (60). In this study, for the first time, demonstrate that 1.0% AKG administration can alleviate the damage of the epithelial barrier caused by the dietary soybean antigen proteins.

Apoptosis is a physiological programmed cell death through the activation of cell intrinsic suicide machinery (61). Apoptosis is a cascade amplifying reaction regulated by a cysteine family of proteases (62). It is well accepted that caspase family proteins is the central regulator of apoptosis (63). We found that the expressions of caspase3 and caspase8 were significantly elevated with FMc group. The expressions of caspase9 was significantly elevated with FMG group, consistent with the results of Peng et al. (64), who showed that soybean antigen protein increased the levels of caspase3 activity and induced piglet intestinal cell apoptosis. Additionally, glycinin or β-conglycinin accelerates enterocyte proliferation, apoptosis and migration for piglets (65). Hence this suggesting that soybean antigen protein causes intestinal damage. However, AKG administration could not alleviate the intestinal apoptosis caused by the dietary soybean antigen proteins in one week. Maybe the longer experimental period might have showed some positive on intestinal apoptosis.

AMPK, a sensor of the availability of intracellular energy, is activated at low energy levels and regulates cellular processes accordingly (66). AMPK is a heterotrimeric Ser/Thr kinase composed of an α, β and γ subunit (67). AMPK is activated by phosphorylation of Thr172 within the activation segment of the KD of the α subunit (68). The β subunits contains a region termed the carbohydrate binding module (68) and allows AMPK to interact with glycogen particles (69). AMPK preserves the level of ATP *via* stimulation of energy producing pathways and suppression of energy-consuming metabolisms (70). Under lowered intracellular ATP levels, AMP or ADP can directly bind to the γ regulatory subunits of AMPK, leading to a conformational change that promotes facilitates the phosphorylation of AMPKα and the activation of AMPK (71). In the current study, the mRNA expression of AMPKγ was significantly elevated and AMPKa was decreased with glycinin. This might be attributed to the fact that soybean antigen protein destroyed the structural stability of the intestine through pro-inflammatory and TJ proteins (54), which required more energy for maintenance, thus increased AMPKγ gene expression. Maintaining the intestinal barrier is energy-consuming, and impairing mitochondria function is associated with intestinal inflammation (72). In addition, AMPK phosphorylation are reported to be involved in promote apoptosis (73). A similar phenomenon was also observed in the study that the expressions of TNF-α, caspase8, and AMPKγ were significantly elevated and claudin7 was significantly decreased in FMG. It indicates that glycinin-induced injure intestine was partially related to the activate of AMPK signaling. In addition, the mRNA level of AMPKγ was decreased in FMGA group. AKG as a source of energy (14), the oxidation of AKG through the tricarboxylic acid cycle can provide large amounts of ATP. AKG stimulates AMPK phosphorylation and oxidation of energy substrates in the intestinal mucosa, thereby enhancing ATP supply and supporting cell function (20). This suggested that AKG increased the intestinal ATP supply partly by down-regulating AMPKγ mRNA expression. It indicates that AKG increases intestinal ATP supply by down-regulating AMPKγ gene expression, but the change of AMP/ATP is not enough to affect AMPK phosphorylation.

AMPK stimulates fatty acid oxidation as a way to increase energy levels. ACC is one of the first proteins identified as a target of AMPK (74). The mRNA level of ACC was increased with the soybean meal and β-conglycinin in this study. ACC is a critical enzyme of the lipogenic pathway (75); simultaneously, it is regulated by AMPK (76). This may indicate that dietary soybean meal and β-conglycinin could increase lipogenesis. In addition, the mTOR, it is also one of the target proteins of the downstream signaling pathway after AMPK (77). It is a limiting step in animal protein synthesis (78). And 4E-BP is one of the mTOR-regulated target sites. In this study, the mRNA level of TOR and 4E-BP were not significantly different with the SM, FMG and FMc for one week. However, studies have showed that β-conglycinin and/or glycinin could decrease the P-TOR protein expression for seven weeks in grass carp (24, 42). The mainly reason is unknown presently, maybe the longer experimental period might have showed some effect of the mTOR signaling pathway.

The fish growth highly depends on the healthy development of the intestine, which can be reflected by the villus height and mucosal thickness (9). Soybean allergy usually induces intestinal inflammatory diseases, characterized by the atrophy and proliferation of crypt villi, which accelerate the apoptosis and migration of intestinal cells (29). In this study, the VH and MT of the proximal intestine, mid intestine, and the distal intestine (except for the FMG group in MI) were decreased and crypt depth of the PI, MI, and DI (except for the SM, FMG group in DI) were increased in SM, FMG and FMc, indicates varying degrees of damage and inflammation. Similar results were also observed in turbot (8) and grass carp (79). Stimulation of antigen-active macromolecules can prompt an immune response that causes atrophy of the intestinal villi, thus damaging the intestinal morphology (9). In addition, dietary AKG has been reported to improve mucosal morphology and function of the intestine (57). In this study, the VH (PI) and MT (DI) were increased and crypt depth (PI) was decreased in FMGA group. The MT (DI) was increased and crypt depth (PI and DI) was decreased in FMcA, which was consistent with studies in piglets (80). In summary, soybean meal, glycinin and β-conglycinin damage the intestinal morphology and AKG improving intestinal morphology

Soybean antigen protein destroys the structural stability of the intestine and causes digestive and absorption disorders (54). Maintaining the intestinal barrier is energy-consuming, and impairing mitochondria function is associated with gut inflammation (72). Under stress, the body’s energy metabolism disorders, ATP content decreased significantly, cell dysfunction or death, leading to damage to intestinal structure and function (81). Citrate synthase, isocitrate dehydrogenase and α-ketoglutarate dehydrogenase are the three rate-limiting enzymes of TCA cycle, which regulate the energy metabolism and biosynthesis of organisms. Citrate synthase can determine the rate of acetyl-CoA into TCA cycle, which is an important indicator of energy metabolism (82). Isocitrate dehydrogenase exists in mitochondria and cytoplasm and catalyzes isocitrate to produce α-ketoglutarate (83). The α-ketoglutarate dehydrogenase system exists in the mitochondrial matrix and catalyzes the oxidative decarboxylation of α-ketoglutarate to produce succinyl coenzyme A and reduced coenzyme I (NADH) (84). Na^+^/K^+^-ATPase mainly exists in the basement membrane of intestinal absorptive cells (85). It produces energy by decomposing ATP to maintain the reverse concentration gradient of sodium and potassium. Its activity can indirectly reflect the absorptive capacity of intestinal mucosa (86). In this study, soybean meal, glycinin and β-conglycinin significantly decreased α-KGDHC, ICD, CS and Na^+^/K^+^-ATPase in DI, indicates that tricarboxylic acid cycle is inhibited and energy production is reduced. The AKG is an important intermediate product in the tricarboxylic acid cycle of organisms, and is also a biosynthetic precursor of glutamic acid, glutamine and arginine in organisms. In addition, glutamine and arginine play an important role in promoting the repair of damaged intestine (87). The AMPKγ, VH (PI), MT (DI), α-KGDHC, ICD, CS, and Na^+^/K^+^-ATPase (PI and MI) were increased in FMGA. The Na^+^/K^+^-ATPase (MI) and MT (DI) were increased in FMcA. These data demonstrate that AKG in enterocytes may beneficially regulate the intracellular endogenous amino acid concentrations *via* TCA cycle (88) and then influence various signaling pathways (89), thereby alleviating the damage intestinal morphology caused by the dietary soybean antigen proteins. In addition, study has shown that AKG can be used as a substrate for intestinal energy metabolism (90). AKG could produce plenty of ATP in the TCA cycle and provide energy for intestinal cell processes (14). Dietary supplementation of AKG can improve the energy metabolism of intestine after soybean antigen protein stimulation.

## 5 Conclusions

In conclusion, dietary soybean meal can compromise the intestine health, and the adverse effects are related to the presence of β-conglycinin and glycinin, especially glycinin. 1.0% AKG may regulate intestinal energy *via* TCA cycle, thereby alleviating the damage intestinal morphology caused by the dietary soybean antigen proteins. Moreover, it is recommended the dietary supplementation of AKG when high levels of soybean meal are used in carp aquafeeds.

## Data availability statement

The original contributions presented in the study are included in the article/supplementary material. Further inquiries can be directed to the corresponding author.

## Ethics statement

This study was conducted following the China’s Guidance of the Care and Use of Laboratory Animals. The experimental protocol was approved by the Committee on the Ethics of Animal Experiments of Huzhou University.

## Author contributions

QL: project administration, writing the original draft preparation, and formal analysis; ZQ and RQ: formal analysis and investigation; FY: writing the manuscript and critical revision; YD and XL: investigation; QX: experiment design, management and manuscript revision. All authors contributed to the article and approved the submitted version.

## References

[1] Li YX Hu HB Liu JT Yang P Zhang YJ Ai QH. Dietary soya allergen β-conglycinin induces intestinal inflammatory reactions, serum-specific antibody response and growth reduction in a carnivorous fish species, turbot scophthalmus maximus l. Aquac Res (2017) 48:4022–37. doi: 10.1016/10.1111/are.13224

[2] Booman M Forster I Vederas JC Groman DB Jones SRM. Soybean meal-induced enteritis in Atlantic salmon (Salmo salar) and Chinook salmon (Oncorhynchus tshawytscha) but not in pink salmon (O. gorbuscha). Aquaculture (2018) 483:238–43. doi: 10.1016/j.aquaculture.2017.10.025

[3] Han FL Wang XD Guo JL Qi CL Xu C Luo Y. Effects of glycinin and β-conglycinin on growth performance and intestinal health in juvenile Chinese mitten crabs (Eriocheir sinensis). Fish Shellfish Immun (2018) 84:269–79. doi: 10.1016/j.fsi.2018.10.013 30300740

[4] Zhang C Rahimnejad S Wang Y Lu K Song K Wang L. Substituting fish meal with soybean meal in diets for Japanese seabass (Lateolabrax japonicus): effects on growth, digestive enzymes activity, gut histology, and expression of gut inflammatory and transporter genes. Aquaculture (2018) 483:173–82. doi: 10.1016/j.aquaculture.2017.10.029

[5] Wang Y Wang L Zhang C Song K. Effects of substituting fishmeal with soybean meal on growth performance and intestinal morphology in orange-spotted grouper (Epinephelus coioides). Aquacult Rep (2017) 5:52–7. doi: 10.1016/j.aqrep.2016.12.005

[6] Zhao JY Xu QY. Influence of soybean meal on intestinal mucosa metabolome and effects of adenosine monophosphate-activated protein kinase signaling pathway in mirror carp (Cyprinus carpio songpu). Front Mar Sci (2022) 9:844716. doi: 10.3389/fmars.2022.844716

[7] He Y Liang J Dong X Liu H Yang Q Zhang S. Soybean β-conglycinin and glycinin reduced growth performance and the intestinal immune defense and altered microbiome in juvenile pearl gentian groupers epinephelus fuscoguttatus♀ × epinephelus lanceolatus♂. Anim Nutr (2022) 9:193–203. doi: 10.1016/j.aninu.2021.11.001 35600546 PMC9092876

[8] Li L Li M Zhu R Yu Z Wang J Duan J. Effects of β-conglycinin on growth performance, antioxidant capacity and intestinal health in juvenile golden crucian carp, carassius auratus. Aquac Res (2019) 50:3231–41. doi: 10.1111/are.14278

[9] Li M Li L YD K Zhu R Yu Z JY W. Effects of glycinin on growth performance, immunity and antioxidant capacity in juvenile golden crucian carp, cyprinus carpio×Carassius auratus. Aquaculture (2020) 51:465–479. doi: 10.1111/are.14390

[10] Duan X Jiang W Wu P Liu Y Jiang J Tan B. Soybean β-conglycinin caused intestinal inflammation and oxidative damage in association with NF-κB, TOR and Nrf2 in juvenile grass carp (Ctenopharyngodon idella): varying among different intestinal segments. Fish Shellfish Immun (2019) 95:105–16. doi: 10.1016/j.fsi.2019.10.021 31610288

[11] Zhang Y Duan X Jiang W Feng L Wu P Liu Y. Soybean glycinin decreased growth performance, impaired intestinal health, and amino acid absorption capacity of juvenile grass carp (Ctenopharyngodon idella). Fish Physiol Biochem (2019) 45:1589–602. doi: 10.1007/s10695-019-00648-z 31256306

[12] Bayliak MM Lushchak VI. Pleiotropic effects of alpha-ketoglutarate as a potential anti-ageing agent. Ageing Res Rev (2021) 66:101237. doi: 10.1016/j.arr.2020.101237 33340716

[13] Hou Y Yao K Wang L Ding B Fu D Liu Y. Effects of α-ketoglutarate on energy status in the intestinal mucosa of weaned piglets chronically challenged with lipopolysaccharide. Brit J Nutr (2011) 106:357–63. doi: 10.1017/S0007114511000249 21342606

[14] Liu SJ Liu HQ Yao K. The antioxidative function of alpha-ketoglutarate and its applications. BioMed Res Int (2018) 2018:1–6. doi: 10.1155/2018/3408467 PMC5884300 29750149

[15] Xu QY Gatlin DM. Effects of alpha-ketoglutarate (AKG) on growth performance and non-specific immunity of juvenile red drum fed diets with low or adequate phosphorus levels. Fish Physiol Biochem (2017) 44:573–82. doi: 10.1007/s10695-017-0454-0 29230593

[16] Zhou ZL. Effects of glycinin and β-conglycinin on growth performance, intestinal halth, metabolic pathway of songpu mirror carp. [master's thesis]. Shanghai: Shanghai Ocean University (2021).

[17] Chen X Xie J Liu Z Yin P Chen M Liu Y. Modulation of growth performance, non-specific immunity, intestinal morphology, the response to hypoxia stress and resistance to aeromonas hydrophila of grass carp (Ctenopharyngodon idella) by dietary supplementation of a multi-strain probiotic. Comp Biochem Phys C (2020) 231:108724. doi: 10.1016/j.cbpc.2020.108724 32061958

[18] Yadav G Meena KM Sahoo AK Das BK Sen R. Effective valorization of microalgal biomass for the production of nutritional fish-feed supplements. J Clean Prod (2020) 243:118697. doi: 10.1016/j.jclepro.2019.118697

[19] Luo J Feng L Jiang W Liu Y Wu P Jiang J. The impaired intestinal mucosal immune system by valine deficiency for young grass carp (Ctenopharyngodon idella) is associated with decreasing immune status and regulating tight junction proteins transcript abundance in the intestine. Fish Shellfish Immun (2014) 40:197–207. doi: 10.1016/j.fsi.2014.07.003 25014314

[20] Lin Y Zhou XQ. Dietary glutamine supplementation improves structure and function of intestine of juvenile jian carp (Cyprinus carpio var. jian). Aquaculture (2006) 256:389–94. doi: 10.1016/j.aquaculture.2006.02.011

[21] Patnaik D Sahu NP Chaudhari A. Effects of feeding raw soybean meal to fry of indian major carp, catla catla, on growth, survival, and protein digestibility. Isr J Aquacult-Bamid (2005) 57:164–74. doi: 10.1016/j.icesjms.2005.07

[22] Yue H Wu J Fu P Ruan R Ye H Hu B. Effect of glutamine supplementation against soybean meal-induced growth retardation, hepatic metabolomics and transcriptome alterations in hybrid sturgeon acipenser baerii♀× a. schrenckii ♂. Aquac Rep (2022) 24:101158. doi: 10.1016/j.aqrep.2022.101158

[23] Yaghoubi M Mozanzadeh MT Marammazi JG Safari O Gisbert E. Dietary replacement of fish meal by soy products (soybean meal and isolated soy protein) in silvery-black porgy juveniles (Sparidentex hasta). Aquaculture (2016) 464:50–9. doi: 10.1016/j.aquaculture.2016.06.002

[24] Duan X Feng L Jiang W Wu P Liu Y Jiang J. The dynamic process of dietary soybean β-conglycinin in digestion, absorption, and metabolism among different intestinal segments in grass carp (Ctenopharyngodon idella). Fish Physiol Biochem (2020) 46:1361–74. doi: 10.1007/s10695-020-00794-9 32221767

[25] Ma YJ Kan GZ Zhang XN Wang YL Zhang W Du HY. Quantitative trait loci (QTL) mapping for glycinin and β-conglycinin contents in soybean (Glycine max l. merr.). J Agric Food Chem (2016) 64:3473. doi: 10.1021/acs.jafc.6b00167 27070305

[26] Zhang JX Guo LY Lin F Jiang WD Kuang SY Liu Y. Soybean β-conglycinin induces inflammation and oxidation and causes dysfunction of intestinal digestion and absorption in fish. PloS One (2014) 8:e58115. doi: 10.1371/journal.pone.0058115 PMC3592885 23520488

[27] Li YX Yang P Zhang YJ Ai QH Xu W Zhang WB. Effects of dietary glycinin on the growth performance, digestion, intestinal morphology and bacterial community of juvenile turbot, scophthalmus maximus l. Aquaculture (2017) 479:125–33. doi: 10.1016/j.aquaculture.2017.05.008

[28] Lin X Jin B Wang H Zhao Y. Effects of diet α-ketoglutarate (AKG) supplementation on the growth performance, antioxidant defense system, intestinal digestive enzymes, and immune response of grass carp (Ctenopharyngodon idellus). Aquacult Int (2020) 28:511–24. doi: 10.1007/s10499-019-00475-2

[29] Wang L Sun ZF Xie WN Peng CL Ding HY Li Y. 11S glycinin up-regulated nlrp-3-induced pyroptosis by triggering reactive oxygen species in porcine intestinal epithelial cells. Front Vet Sci (2022) 9:890978. doi: 10.3389/fvets.2022.890978 35782549 PMC9240605

[30] Han F Xu C Qi C Lin Z Li E Wang C. Sodium butyrate can improve intestinal integrity and immunity in juvenile Chinese mitten crab (Eriocheir sinensis) fed glycinin. Fish Shellfish Immun (2020) 102:400–11. doi: 10.1016/j.fsi.2020.04.058 32371256

[31] Ameur WB Lapuente DJ Megdiche Y Barhoumi B Trabelsi S Camps L. Oxidative stress, genotoxicity and histopathology biomarker responses in mullet (Mugil cephalus) and sea bass (Dicentrarchus labrax) liver from bizerte lagoon (Tunisia). Mar pollut Bull (2012) 64:241–51. doi: 10.1016/j.marpolbul.2011.11.026 22206722

[32] Yi LY Liu JW Yang HJ Mo AJ Zhai YX Wang SR. Effects of dietary glycinin on oxidative damage, apoptosis and tight junction in the intestine of juvenile hybrid yellow catfish, pelteobagrus fulvidraco ♀× pelteobaggrus vachelli ♂. Int J Mol Sci (2022) 23:11198. doi: 10.3390/ijms231911198 36232502 PMC9570327

[33] Gu M Pan S Li Q Qi Z Deng W Bai N. Protective effects of glutamine against soy saponins-induced enteritis, tight junction disruption, oxidative damage and autophagy in the intestine of scophthalmus maximus l. Fish Shellfish Immun (2021) 114:49–57. doi: 10.1016/j.fsi.2021.04.013 33887442

[34] Zhang F Wang X Wang W Li LJ. Glutamine reduces TNF-α by enhancing glutathione synthesis in lipopolysaccharide-stimulated alveolar epithelial cells of rats. Inflammation (2008) 31:344–50. doi: 10.1007/s10753-008-9084-0 18807160

[35] Ali R Mittal G Sultana S Bhatnagar A. Ameliorative potential of alpha-ketoglutaric acid (AKG) on acute lung injuries induced by ammonia inhalation in rats. Exp Lung Res (2012) 38:435–44. doi: 10.3109/01902148.2012.721859 22978367

[36] Liu S He L Yao K. Impact of nutritional and environmental factors on inflammation, oxidative stress, and the microbiome. BioMed Res Int (2018) 2018:3408467. doi: 10.1155/2018/5606845 30151385 PMC6091408

[37] Maria B Nadia B Volodymyr L. Growth on alpha-ketoglutarate increases oxidative stress resistance in the yeast saccharomyces cerevisiae. Int J Food Microbiol (2017) 2017:5792192. doi: 10.1155/2017/5792192 PMC5244014 28154578

[38] Francis G Makkar HPS Becker K. Antinutritional factors present in plant-derived alternate fish feed ingredients and their effects in fish. Aquaculture (2001) 199:197–227. doi: 10.1016/S0044-8486(01)00526-9

[39] Cho K Adamson LK Greenhalgh DG. Parallel self-induction of TNF-α and apoptosis in the thymus of mice after burn injury. J Surg Res (2001) 98:9–15. doi: 10.1006/jsre.2001.6157 11368531

[40] Huising MO Stet R Savelkoul H Kemenade VV. The molecular evolution of the interleukin-1 family of cytokines; IL-18 in teleost fish. Dev Comp Immunol (2004) 28:395–413. doi: 10.1016/j.dci.2003.09.005 15062640

[41] Dinarello CA. Proinflammatory cytokines. Chest (2000) 118:503–8. doi: 10.1378/chest.118.2.503 10936147

[42] Zhang YL Duan XD Feng L Jiang WD Zhou XQ. Soybean glycinin impaired immune function and caused inflammation associated with PKC-ζ/NF-κb and mTORC1 signaling in the intestine of juvenile grass carp (Ctenopharyngodon idella). Fish Shellfish Immun (2020) 106:393–403. doi: 10.1016/j.fsi.2020.08.008 32800984

[43] Wang K Feng L Jiang W Wu P Liu Y Jiang J. Dietary gossypol reduced intestinal immunity and aggravated inflammation in on-growing grass carp (Ctenopharyngodon idella). Fish Shellfish Immun (2019) 86:814–31. doi: 10.1016/j.fsi.2018.12.014 30543935

[44] Zhu R Li L Li M Yu Z Wu LF. Effects of dietary glycinin on the growth performance, immunity, hepatopancreas and intestinal health of juvenile rhynchocypris lagowskii dybowski. Aquaculture (2021) 544:737030. doi: 10.1016/j.aquaculture.2021.737030

[45] Chen S Bin P Ren W Gao W Yin Y. Alpha-ketoglutarate (AKG) lowers body weight and affects intestinal innate immunity through influencing intestinal microbiota. Oncotarget (2017) 8:38184–92. doi: 10.18632/oncotarget.17132 PMC5503525 28465471

[46] Yao J Kong C Hua X Yang J Liu T Wang G. T1R1 expression in obscure puffer (Takifugu fasciatus) is associated with effect of dietary soybean antigenic protein on intestinal health. Aquaculture (2019) 501:202–12. doi: 10.1016/j.aquaculture.2018.11.010

[47] Harsha WTF Kalandarova E Mcnutt P Irwin R Noel J. Nutritional supplementation with transforming growth factor-beta, glutamine, and short chain fatty acids minimizes methotrexate-induced injury. J Pediatr Gastr Nutr (2006) 42:53–8. doi: 10.1097/01.mpg.0000189136.06151.7a 16385254

[48] Yu YB Li YQ. Enteric glial cells and their role in the intestinal epithelial barrier. World J Gastroenterol (2014) 32:11273–80. doi: 10.3748/wjg.v20.i32.11273 PMC4145765 25170211

[49] Anderson JM Itallie CMV. Physiology and function of the tight junction. Csh Perspect Biol (2009) 1:a002584. doi: 10.1101/cshperspect.a002584 PMC2742087 20066090

[50] Itallie C Anderson JM. Architecture of tight junctions and principles of molecular composition. Semin Cell Dev Biol (2014) 36:157–65. doi: 10.1016/j.semcdb.2014.08.011 PMC4254347 25171873

[51] Wang Y Mumm JB Herbst R Kolbeck R Wang Y. IL-22 increases permeability of intestinal epithelial tight junctions by enhancing claudin-2 expression. J Immunol (2017) 199:3316–25. doi: 10.4049/jimmunol.1700152 28939759

[52] Takiishi T Fenero CIM Camara NOS. Intestinal barrier and gut microbiota: shaping our immune responses throughout life. Tiss Barr (2017) 5:e1373208. doi: 10.1080/21688370.2017.1373208 PMC5788425 28956703

[53] Zhao Y Qin G Han R Wang J Zhang X Liu D. β-conglycinin reduces the tight junction occludin and ZO-1 expression in IPEC-J2. Int J Mol Sci (2014) 15:1915–26. doi: 10.3390/ijms15021915 PMC3958829 24473141

[54] Jiang WD Hu K Zhang JX Liu Y Jiang J Wu P. Soyabean glycinin depresses intestinal growth and function in juvenile jian carp (Cyprinus carpio var jian): protective effects of glutamine. Br J Nutr (2015) 114:1569–83. doi: 10.1017/S0007114515003219 26349522

[55] He LQ Zhou XH Wu ZP Feng YZ Liu D Li TJ. Glutamine in suppression of lipopolysaccharide-induced piglet intestinal inflammation: the crosstalk between AMPK activation and mitochondrial function. Anim Nutr (2022) 3:11. doi: 10.1016/j.aninu.2022.03.001 PMC9149014 35663373

[56] Bruewer M Luegering A Kucharzik T Parkos CA Madara JL Hopkins AM. Proinflammatory cytokines disrupt epithelial barrier function by apoptosis-independent mechanisms. J Immunol (2003) 171:6164–72. doi: 10.4049/jimmunol.171.11.6164 14634132

[57] Hou Y Wang L Ding B Liu Y Zhu H Liu J. Alpha-ketoglutarate and intestinal function. Front Biosci (2011) 16:1186–96. doi: 10.2741/3783 21196226

[58] Wang LS Fan Z Zhang YY Wu D Li JN Xu QY. Effect of phosphorus on growth performance, intestinal tight junctions, Nrf2 signaling pathway and immune response of juvenile mirror carp (Cyprinus carpio) fed different α-ketoglutarate levels. Fish Shellfish Immun (2022) 120:271–9. doi: 10.1016/j.fsi.2021.11.040 34863945

[59] Liu Y Chen Z Dai J Yang P Hu H Ai Q. The protective role of glutamine on enteropathy induced by high dose of soybean meal in turbot, scophthalmus maximus l. Aquaculture (2018) 497:510–9. doi: 10.1016/j.aquaculture.2018.08.021

[60] Rezaei R Knabe DA Tekwe CD. Dietary supplementation with monosodlum glutamate is safe and improves growth performance in postweaning pigs. Amino Acids (2013) 44:911–23. doi: 10.1007/s00726-012-1420-x 23117836

[61] Ning HQ Li YQ Tian QW Wang ZS Mo HZ. The apoptosis of staphylococcus aureus induced by glycinin basic peptide through ROS oxidative stress response. Lwt-Food Sci Technol (2018) 99:62–8. doi: 10.1016/j.lwt.2018.09.028

[62] Wang H Wang A Wang X Zeng X Xing H. AMPK/PPAR-γ/NF-κB axis participates in ROS-mediated apoptosis and autophagy caused by cadmium in pig liver. Environ pollut (2022) 294:118659. doi: 10.1016/j.envpol.2021.118659 34896222

[63] Birkinshaw RW Czabotar PE. The BCL-2 family of proteins and mitochondrial outer membrane permeabilisation. Semin Cell Dev Biol (2017) 72:152–62. doi: 10.1016/j.semcdb.2017.04.001 28396106

[64] Peng CL Cao CM Shu MC Tang YS Wang XB. Soybean glycinin- and β-conglycinin-induced intestinal damage in piglets *via* the p38/JNK/NF-κB signaling pathway. J Agr Food Chem (2018) 66:9534–41. doi: 10.1021/acs.jafc.8b03641 30139257

[65] Zhao Y Qin GX Sun ZW Zhang B Wang T. Effects of glycinin and β-conglycinin on enterocyte apoptosis, proliferation and migration of piglets. Food Arg Immunol (2010) 21:209–18. doi: 10.1080/09540101003596644

[66] Schultze SM Hemmings BA Markus N Oliver T. PI3K/AKT, MAPK and AMPK signalling: protein kinases in glucose homeostasis. Expert Rev Mol Med (2012) 14:e1. doi: 10.1017/S1462399411002109 22233681

[67] Carles C Auwerx J. AMP-activated protein kinase and its downstream transcriptional pathways. Cell Mol Life Sci (2010) 67:3407–23. doi: 10.1007/s00018-010-0454-z PMC3622821 20640476

[68] Carling D. AMPK signalling in health and disease. Environ Exp Bot (2017) 141:31–7. doi: 10.1016/j.ceb.2017.01.005 28232179

[69] Hudson ER Pan DA James J Lucocq JM Hawley SA Green KA. A novel domain in AMP-activated protein kinase causes glycogen storage bodies similar to those seen in hereditary cardiac arrhythmias. Curr Biol (2003) 13:861–6. doi: 10.1016/S0960-9822(03)00249-5 12747836

[70] Kerui F Ling L Qing A Jingyuan W Jie D Gang L. Lipopolysaccharide-induced dephosphorylation of AMPK-activated protein kinase potentiates inflammatory injury via repression of ULK1-dependent autophagy. Front Immunol (2018) 9:1464. doi: 10.3389/fimmu.2018.01464 29988556 PMC6026648

[71] Mihaylova MM Shaw RJ. The AMPK signalling pathway coordinates cell growth, autophagy and metabolism. Nat Cell Biol (2011) 13:1016–23. doi: 10.1038/ncb2329 PMC3249400 21892142

[72] Ho GT Aird RE Liu B Boyapati RK Kennedy NA Dorward DA. MDR1 deficiency impairs mitochondrial homeostasis and promotes intestinal inflammation. Mucosal Immunol (2018) 11:120–30. doi: 10.1038/mi.2017.31 PMC5510721 28401939

[73] Zhang MH Fang XS Guo JY Jin Z. Effects of AMPK on apoptosis and energy metabolism of gastric smooth muscle cells in rats with diabetic gastroparesis. Cell Biochem Biophys (2019) 77:165–77. doi: 10.1007/s12013-019-00870-9 30968342

[74] Lepropre S Kautbally S Octave M Ginion A Onselaer MB Steinberg GR. AMPK-ACC signaling modulates platelet phospholipids and potentiates thrombus formation. Blood (2018) 132:1180–92. doi: 10.1182/blood-2018-02-831503 PMC6238154 30018077

[75] Sim A Hardie DG. The low activity of acetyl-CoA carylase in basal and glucagon-stimulated hepatocytes is due to phosphorylation by the AMP-activated protein kinase and not cyclic AMP-dependent protein kinase. FEBS Lett (1988) 233:294–8. doi: 10.1016/0014-5793(88)80445-9 2898386

[76] Park H. Coordinate regulation of malonyl-CoA decarboxylase,sn-glycerol-3-phosphate acyltransferase, and acetyl-CoA carboxylase by AMP-activated protein kinase in rat tissues in response to exercise. J Biol Chem (2002) 277:32571–7. doi: 10.1074/jbc.M201692200 12065578

[77] Saxton RA Sabatini DM. mTOR signaling in growth, metabolism, and disease. Cell (2017) 168:960–76. doi: 10.1016/j.cell.2017.02.004 PMC5394987 28283069

[78] Preiss T Hentze MW. Starting the protein synthesis machine: eukaryotic translation initiation. Bioessays (2003) 25:1201–11. doi: 10.1002/bies.10362 14635255

[79] Duan X Feng L Jiang W Wu P Liu Y Kuang S. Dietary soybean β-conglycinin suppresses growth performance and inconsistently triggers apoptosis in the intestine of juvenile grass carp (Ctenopharyngodon idella) in association with ROS-mediated MAPK signalling. Aquacult Nutr (2019) 25:770–82. doi: 10.1111/anu.12895

[80] Hu QZ Hou YQ Ding BY Zhu HL Liu YL Wang M. Effects of α-ketoglutarate on histological morphology and function of small intestine in piglets. Chin J Anim Nutr (2008) 20:662–7.

[81] Coyne VE. The importance of ATP in the immune system of molluscs. Invert Suriviv J (2011) 8:48–55.

[82] Ren M Yang X Bie J Wang Z Liu M Li Y. Citrate synthase desuccinylation by SIRT5 promotes colon cancer cell proliferation and migration. Biol Chem (2020) 401:1031–9. doi: 10.1515/hsz-2020-0118 32284438

[83] Lee JH Go YH Kim DY Lee SH Kim OH Jeon YH. Isocitrate dehydrogenase 2 protects mice from high-fat diet-induced metabolic stress by limiting oxidative damage to the mitochondria from brown adipose tissue. Exo Mol Med (2020) 52:238–52. doi: 10.1038/s12276-020-0379-z PMC7062825 32015410

[84] Burch JS Marcero JR Maschek JA Cox JE Jackson LK Medlock AE. Glutamine *via* α-ketoglutarate dehydrogenase provides succinyl-CoA for heme synthesis during erythropoiesis. Blood (2018) 132:987–98. doi: 10.1182/blood-2018-01-829036 PMC6128084 29991557

[85] Saha P Manoharan P Arthur S Sundaram S Sundaram U. Molecular mechanism of regulation of villus cell Na-K-ATPase in the chronically inflamed mammalian small intestine. Bba - Biomembranes (2015) 1848:702–11. doi: 10.1016/j.bbamem.2014.11.005 25462166

[86] Veillette PA Young G. Temporal changes in intestinal na^+^, k^+^-ATPase activity and *in vitro* responsiveness to cortisol in juvenile chinook salmon. Comp Biochem Phys A (2004) 138:297–303. doi: 10.1016/j.cbpb.2004.04.007 15313483

[87] Wu G Meininger CJ Knabe DA Baze FW Rhoads JM. Arginine nutrition in development, health and disease. Curr Opin Clin Nutr Metab Care (2000) 3:59–66. doi: 10.1016/j.jnutbio.2003.11.010 10642085

[88] Xue H Sufit AJ Wischmeyer PE. Glutamine therapy improves outcome of in vitro and in vivo experimental colitis models. Jpen-Parenter enter (2011) 35:188–97. doi: 10.1177/0148607110381407 21378248

[89] He LQ Huang N Li H Tian JQ Zhou XH Li TJ. AMPK/alpha-ketoglutarate axis regulates intestinal water and ion homeostasis in young pigs. J Agric Food Chem (2017) 65:2287–98. doi: 10.1021/acs.jafc.7b00324 28241728

[90] Wu GY. Intestinal mucosal amino acid catabolism. J Nutr (1998) 128:1249–52. doi: 10.1038/sj.ijo.0800681 9687539

